# Immune Assisted Tissue Engineering via Incorporation of Macrophages in Cell-Laden Hydrogels Under Cytokine Stimulation

**DOI:** 10.3389/fbioe.2018.00108

**Published:** 2018-08-20

**Authors:** Julien Barthes, Camille Dollinger, Celine B. Muller, Urmas Liivas, Agnes Dupret-Bories, Helena Knopf-Marques, Nihal E. Vrana

**Affiliations:** ^1^PROTiP Medical, Strasbourg, France; ^2^INSERM UMR 1121, Strasbourg, France; ^3^Protobios LLC, Tallinn, Estonia; ^4^Institut Claudius Regaud, Institut Universitaire du Cancer Toulouse-Oncopole, Toulouse, France; ^5^Faculté de Chirurgie Dentaire, Université de Strasbourg, Strasbourg, France

**Keywords:** macrophage, encapsulation, hydrogels, tissue engineering, cytokines, co-culture

## Abstract

The function of soft tissues is intricately linked to their connections with the other systems of the body such as circulation, nervous system, and immune system. The presence of resident macrophages in tissues provides a means to control tissue homeostasis and also a way to react to the physical/biological insults and tissue damage. Thus, incorporation of resident macrophage like phenotype-controlled macrophages in engineered tissues can improve their fidelity as model tissues and also improve their rate of integration and facilitate the resolution of inflammation for regenerative medicine applications. Herein, we demonstrate two potential ways to immunoassist the remodeling process of engineered soft tissues in three-dimensional (3-D) gelatin based hydrogels containing fibroblasts and/or endothelial cells: (i) with supplementation of interleukin-4 (IL-4) in the presence of macrophages and (ii) in tri-culture via naive monocytes or differentiated macrophages. The presence of IL-4 had a proliferative effect on fibroblasts, with a significant boosting effect on proliferation and cytokine secretion in the presence of differentiated macrophages with an upregulation of activin, interleukin-1 receptor antagonist (IL-1RA), tumor necrosis factor alpha (TNF-α), and interleukin-1 beta (IL-1β), creating a more stimulating microenvironment. The addition of IL-4 in endothelial cell/macrophage co-culture configuration improved the organization of the sprout-like structures, with a boost in proliferation at day 1 and with an upregulation of IL-6 and IL-1RA at the earliest stage in the presence of differentiated macrophages creating a favorable microenvironment for angiogenesis. In tri-culture conditions, the presence of monocytes or macrophages resulted in a denser tissue-like structure with highly remodeled hydrogels. The presence of differentiated macrophages had a boosting effect on the angiogenic secretory microenvironment, such as IL-6 and IL-8, without any additional cytokine supplementation. The presence of fibroblasts in combination with endothelial cells also had a significant effect on the secretion of angiopoietin. Our results demonstrate that incorporation of macrophages in a resident macrophage function and their phenotype control have significant effects on the maturation and cytokine microenvironment of 3-D multiple cell type-laden hydrogels, which can be harnessed for better integration of implantable systems and for more physiologically relevant *in vitro* tissue models with an immune component.

**Graphical Abstract:**
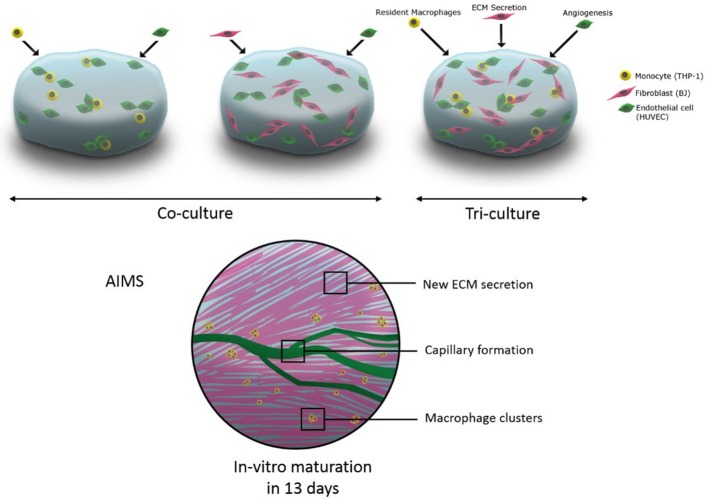
The schematic representation of utilisation of tri-culture systems including macrophages in hydrogels for immune assisted tissue engineering.

## Introduction

Macrophages possess an important role in regulating multiple tissue repair processes because of their direct relation to all stages of tissue healing through their phenotypic plasticity (Spiller and Koh, 2017). Thus, for regenerative medicine and tissue engineering applications, harnessing the pro-healing properties of macrophages has a strong potential for facilitated *in vitro* tissue maturation or improved *in vivo* integration and vascularization.

In a multifactorial n-dimension polarization space, the macrophage polarization has been generally described in a simplified spectrum of M1 (pro-inflammatory) and M2 (anti-inflammatory) macrophages with subgroups (M2a, M2c, etc.) (Mantovani et al., 2005). A recent study on the gene expression and protein secretion profiles of different macrophage phenotypes for angiogenesis *in vitro* has shown that all phenotypes support angiogenesis in different ways. M1 and M2c induced endothelial cell sprouting, whereas M2a macrophages promoted anastomosis (Spiller et al., 2014). In tissue repair, the chronological appearance of M1 and M2 macrophage phenotypes correspond to inflammation or initiation of healing process and stabilization and tissue maturation, respectively (Porcheray et al., 2005; Rostam et al., 2016; Cha et al., 2017). A recent co-culture study with a three-dimensional (3-D) polyethylene glycol (PEG)-based system has shown the influence of macrophages on angiogenesis and vasculogenesis. The macrophages were capable of influencing vessel formation within this system. Furthermore, macrophages can also enhance tubule formation by altering the morphology of endothelial cells and by associating with them in a bridging and pericyte-like manner (Moore et al., 2017).

In regard to wound healing and regeneration, harnessing the host macrophages to enhance the differentiation of delivered cells has become a good strategy for regenerative medicine. Niu et al. (2017) recently designed a new acetyl *Bletilla striata* polysaccharide (acBSP) polymer coating, which was able to promote the activation of tissue macrophages at the host-scaffold interface to secrete pro-regenerative cytokines that can enhance the osteogenesis of the mesenchymal stem cells inside the scaffold (Niu et al., 2017).

Macrophage polarization is a strong component in many biological events such as bacterial clearance, wound healing, tumor development, and foreign body response. The phenotypic plasticity of macrophages and their fast reversion between different polarization states enable them to react to adverse events in a timely and effective manner. One of the most common inducers of M2 differentiation is interleukin-4 (IL-4) stimulation (Martinez et al., 2009). A product of T-cells and mast cells, IL-4, has been shown to induce M2 related cellular behavior *in vitro* [high CD206 expression, low levels of tumor necrosis factor alpha (TNF-α), IL-1 beta (IL-1β) release with high levels of IL-1 receptor antagonist (IL-1RA), and chemokine (C-C motif) ligand 18 (CCL18) release] (Martinez-Santibañez and Lumeng, 2014). Moreover, IL-4 is known to induce collagen secretion by fibroblasts (Fertin et al., 1991) and has been shown to induce angiogenesis by upregulating vascular cell adhesion molecule-1 (VCAM-1) expression *in vitro* (Fukushi et al., 2000). Even though IL-4 is not a cytokine produced by macrophages, it has been recognized as one of the main inducers of M2 macrophage polarization. Beyond its role in M2 macrophage polarization, IL-4 has also been known to induce collagen secretion by fibroblasts and angiogenesis, which makes it a good candidate to develop immunoassisted engineered tissues. In fact, this cytokine has an effect on the three relevant cell types that are used to develop immunoassisted tissues: immune cells (monocytes and macrophages), fibroblasts, and endothelial cells. Nevertheless, this effect needs to be tightly controlled as IL-4 is also active in fibrosis, tumor formation via tumor-associated macrophages, and also in atherosclerosis. Thus, it is important to provide such potent cytokines in a proper 3-D extracellular matrix (ECM) microenvironment. One potential way of harnessing IL-4 stimulated macrophage activity is their direct incorporation into 3-D hydrogel structures for a given tissue (Vrana, 2016).

Over the last few years, the demand for the development of new strategies for the repair of diseased and damaged tissues has increased (Brodbeck et al., 2002; Anderson et al., 2008; Brown and Badylak, 2013). One potential way is the delivery of cells to the damaged tissue, which involves cell encapsulation in biopolymer gels such as alginate, collagen, fibrin, hyaluronic acid, and gelatin, which are described as promising materials for tissue engineering of cartilage, bone, ligament, tendon, skin, and blood vessels in the presence of growth factors and cytokines (Hunt and Grover, 2010). In particular, gelatin which is formed from hydrolysis of collagen is widely used in medical applications because of its biodegradable nature (Ikada and Tabata, 1996; Tabata and Ikada, 1998; Miyoshi et al., 2005). However, gelatin is soluble in aqueous solution at 37°C and for biomedical applications it must be crosslinked to improve the mechanical and thermal stability. The crosslinking can be done by chemical crosslinkers [glutaraldehyde (Bigi et al., 2001), 1-ethyl-3-(3-dimethylaminopropyl)-carbodiimide/N-hydroxysuccinimide (EDC/NHS) (Liang et al., 2004), or genipin (Bigi et al., 2002)], photocrosslinking [methacrylated gelatin by UV irradiation (Nichol et al., 2010)], or by enzymatically catalyzed reactions [transglutaminase (TGA) (De Carvalho and Grosso, 2004; McDermott et al., 2004) or tyrosinase (Jus et al., 2012)]. Studies conducted with endothelial cells demonstrated that, once they were encapsulated in gelatin methacryloyl (GelMA) hydrogels, the cells readily bind to, proliferate, elongate, and migrate in the matrix (Nichol et al., 2010). These results revealed that gelatin based materials, such as GelMA, act as an attractive material for creating cell-laden microtissues (Ngo and Harley, 2017; Prakash Parthiban et al., 2017). Among the different crosslinking methods discussed, using TGA crosslinking reaction has the most advantages because of the absence of toxic components, such as crosslinking agents or photoinitiators. Transglutaminase catalyzes the formation of amide crosslink between carboxamide (RCONH2) from glutamine and primary amine functionalities from lysine (Orban et al., 2004).

Vascularization of the engineered constructs with the creation of functional blood vessels is one of the key parameters of tissue engineering and regenerative medicine. Blood vessels provide nutrients, oxygen, and also eliminate the waste, which are crucial for tissue maturation (Jain et al., 2005). It has been shown that an engineered tissue construct without vascularization is limited to about 100–200 μm thickness, and this is the reason why a vascularization strategy must be considered with all thick engineered tissues. Different strategies have been envisioned to vascularize tissue constructs, such as introduction of cellular components (endothelial cells as main components), to assist the remodeling of tissues necessary for the creation of blood vessels or introduction of channels in the scaffold using, for example, 3-D printing techniques with sacrificial network which will accelerate blood vessel formation (Kolesky et al., 2016). In all these strategies, one of the main components is the cellular component, and to achieve fully functional vascular network many cell types present in soft tissues are required. This necessitates a co-culture system and the study of interactions between all these cells is necessary to better understand this process. Moreover, the major limitation with all these strategies is the time required to achieve the vascularization. To solve this problem, the introduction of immune components in the construct, such as macrophages, could harness the inflammation and promote the healing process with a faster vascularization, even for thick engineered tissue constructs (Spiller et al., 2014; Tattersall et al., 2016; Moore et al., 2017).

Recently, we have demonstrated the effect of encapsulated macrophages on incoming cells in an *in vitro* model, where the cytokine microenvironment had a significant effect on cell behavior and subsequent cytokine secretion profiles (Dollinger et al., 2017). However, the co-encapsulation of naive or activated macrophages in a 3-D environment with connective tissue cells has not been widely studied. Some recent work, demonstrating the ability of macrophages to take part in a pericyte-like manner for capillary sprouting *in vitro* (Moore et al., 2017), suggests that such an approach can be potentially beneficial for tissue maturation in regenerative medicine applications.

Our aim is to incorporate inflammation related signals into the engineered tissues in the form of different cell types co-encapsulated in hydrogels with the presence of cytokines (IL-4, selected to induce a resident macrophage like phenotype) and monocytes and macrophages (to contribute to the remodeling and an artificial homeostasis in the hydrogel via paracrine and cell-cell contact effects). As a proof of concept, the effects of immune cell inclusion and IL-4 presence were tested in a model containing a connective tissue cell type (fibroblasts) and vascular endothelial cells for potential vascularization in gelatin hydrogels crosslinked with TGA. In a previous study, co-culture and tri-culture experiments have been performed with primary osteoblasts, endothelial cells, and THP-1 derived macrophages using a ratio of 1/10 (macrophages/osteoblasts) and 1/6 (macrophages/endothelial cells); in order to get sufficient number of macrophages to remodel our cell-laden hydrogel, we have decided to perform all our experiments using a single ratio of 1/6 (fibroblasts or endothelial cells/macrophages) (Dohle et al., 2014).

## Materials and methods

### Materials

Gelatin type A from porcine skin (Mw = 5–10 × 10^4^ Da, G-1890) was purchased from Sigma-Aldrich (Saint-Quentin-Fallavier, France). Interleukin-4 (recombinant human interleukin-4, *E. coli*-derived, C-61421), human umbilical vein endothelial cells (HUVEC) (Promocell® C-12200), endothelial cell growth medium (C-22010), apoptotic/necrotic/healthy cells detection kit (PK-CA707-30018), fluorimetric cell viability kit I (Resazurin, PK-CA707-30025-1), and 4′,6-diamidino-2-phenylindole (DAPI) (PK-CA707-40009) were purchased from Promocell (Heidelberg, Germany). Human monocytic cell line (THP-1, ATCC® TIB-202) and human fibroblast cells (BJ2, ATCC® CRL-2522) were purchased from ATCC (Manassas, US) in frozen form. RPMI-1640 and DMEM media, Dulbecco's phosphate buffered saline (DPBS), fetal bovine serum (FBS), 0.05% trypsin/0.02% EDTA, TripLE™ Express (1x), β-mercaptoethanol, Cell tracker™ (C-2925), and Vybrant cell adhesion assay kit (V-13181) were obtained from Life Technologies (Carlsbad, USA). Microbial TGA (Mw = 3.8 × 10^4^ Da) was kindly provided by Ajinomoto Inc. (Tokyo, Japan).

### Methods

#### Cell lines

The THP-1 cells were cultured in RPMI 1640 GlutaMAX supplemented with 10% FBS, 1% penicillin/streptomycin, 0.2% fungizone, and 0.05 mM 2-mercaptoethanol. To differentiate from monocytes to macrophages, cells were treated with 50 ng of phorbol myristate acetate (PMA), dissolved in medium (RPMI 1640 without 2-mercaptoethanol), for 24 h at 37°C and 5% CO_2_. Unattached cells were removed after washing with DPBS. Phorbol myristate acetate activated THP-1 cells were detached using TripLE ™ Express (1x), centrifuged, and resuspended in medium (without 2-mercaptoethanol).

The BJ2 cells were cultured in RPMI 1640 (Gibco Life Technologies, USA) supplemented with 10% FBS, 1% penicillin/streptomycin, 0.2% fungizone at 37°C in a 5% CO_2_ atmosphere. Prior to seeding, cells were harvested using 0.05% trypsin/0.02% EDTA, centrifuged, and resuspended in medium.

Human umbilical vein endothelial cells were used from passages between 4 and 8. The culture media used were endothelial cell growth medium supplemented with Supplement Mix C-39215 (mainly composed of heparin, hydrocortisone, fetal calf serum, basic fibroblast growth factor, epithelial growth factor, and endothelial cell growth supplement) and 1% penicillin/streptomycin. Prior to seeding, cells were harvested using 0.05% trypsin/0.02% EDTA, centrifuged, and resuspended in medium.

#### Cell encapsulation in gelatin hydrogel

For the encapsulation experiments, the gelatin solution was prepared in a 6% w/v gelatin type A/ MilliQ water at 37°C until complete dissolution; microbial TGA solution was prepared in a 20% w/v in PBS. The cell encapsulation was done with different cell types: monocytes (naive THP-1), human fibroblasts (BJ2), HUVECs, and macrophages (activated THP-1) cells. The cells were encapsulated in gelatin solution with a concentration of 1 × 10^6^ cells mL^−1^ for naive and activated THP-1 and 6 × 10^6^ cells mL^−1^ for HUVEC and fibroblasts cells. A 50 μL aliquot of cells encapsulated in gelatin solution was mixed with 10 μL TGA solution to get homogeneous crosslinking effect, and the hydrogel was then kept in incubator at 37°C for at least 15 min.

For the conditions with IL-4 supplementation, supernatant was supplemented with 10 ng mL^−1^ IL-4 each time the medium was changed (days 3, 7, and 10). The concentration of IL-4 (10 ng mL^−1^) was chosen according to our previous study, where macrophages were successfully polarized into M2 phenotype using this concentration (Riabov et al., 2017).

**Scheme 1:**
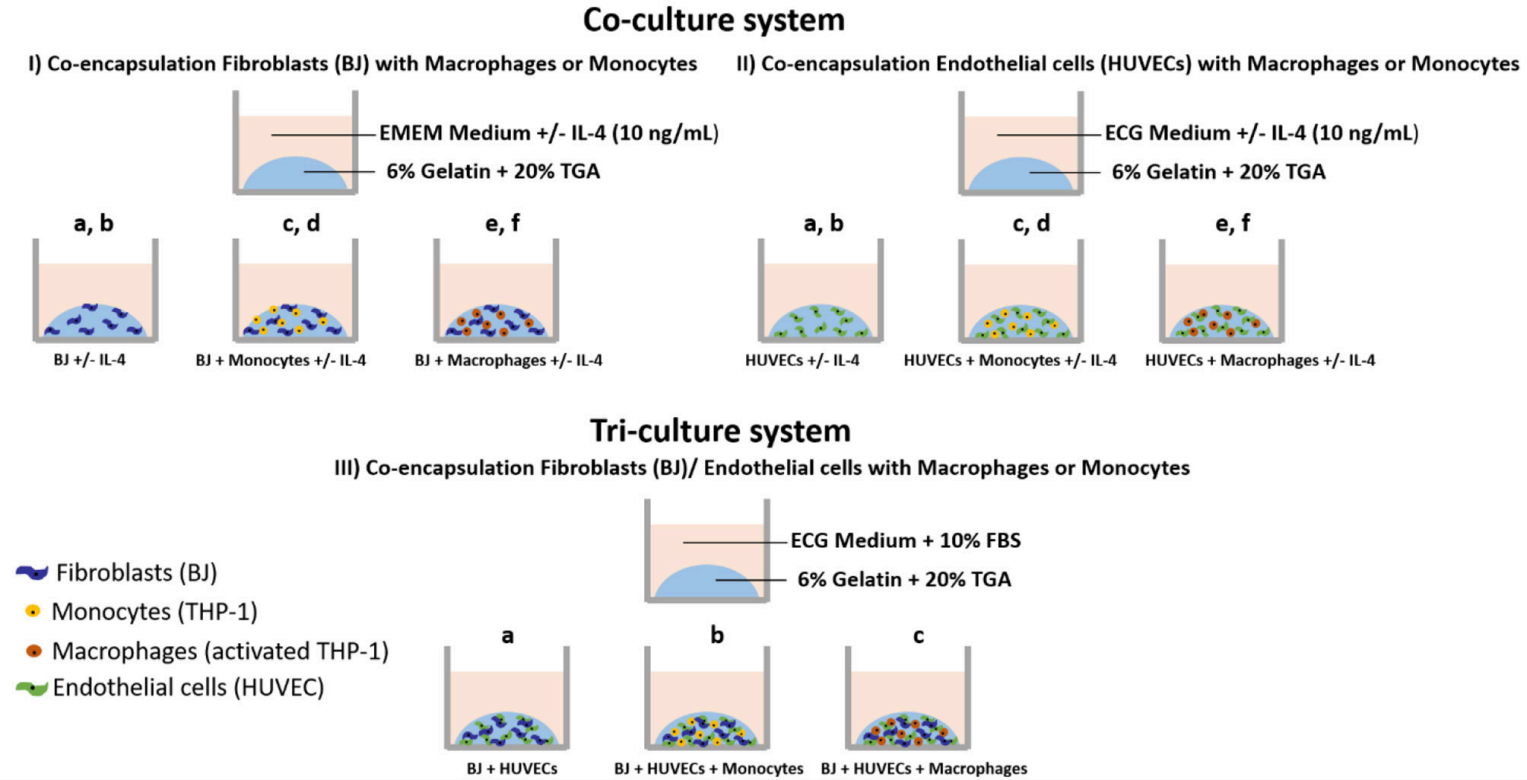
Cell encapsulation conditions. **(I)** Co-culture experiments with fibroblasts: **(Ia)** Fibroblasts alone **(BJ2)**; **(Ib)** Fibroblasts alone + IL-4 supplementation **(BJ2** + **IL-4)**; **(Ic)** Fibroblasts + Monocytes **(BJ2** + **Monocytes)**; **(Id)** Fibroblasts + Monocytes + IL-4 supplementation **(BJ2** + **Monocytes** + **IL-4)**; **(Ie)** Fibroblasts + Macrophages **(BJ2** + **Macrophages)**; and **(If)** Fibroblasts + Macrophages + IL-4 supplementation **(BJ2** + **Macrophages** + **IL-4)**. **(II)** Co-culture experiments with endothelial cells: **(IIa)** Endothelial cells alone **(HUVECs)**; **(IIb)** Endothelial cells alone + IL-4 supplementation **(HUVECs** + **IL-4)**; **(IIc)** Endothelial cells + Monocytes **(HUVECs** + **Monocytes)**; **(IId)** Endothelial cells + Monocytes + IL-4 supplementation **(HUVECs** + **Monocytes** + **IL-4)**; **(IIe)** Endothelial cells + Macrophages **(HUVECs** + **Macrophages)**; and **(IIf)** Endothelial cells + Macrophages + IL-4 supplementation **(HUVECs** + **Macrophages** + **IL-4)**. **(III)** Tri-culture experiments: **(IIIa)** Fibroblasts + Endothelial cells **(BJ** + **HUVECs)**; **(IIIb)** Fibroblasts + Endothelial cells + Monocytes **(BJ2** + **HUVECs** + **Monocytes)**; and **(IIIc)** Fibroblasts + Endothelial cells + Macrophages **(BJ2** + **HUVECs** + **Macrophages)**.

The experiments were done with encapsulated cells in gelatin hydrogel under different conditions, as shown in Scheme 1:

- *In EMEM medium (supplemented with 10% FBS) with or without IL-4 (10 ng mL*^−1^*):* Fibroblasts alone, **BJ2** (Scheme Ia); Fibroblasts alone + IL-4 supplementation, **BJ2** + **IL-4** (Scheme Ib); Fibroblasts + Monocytes, **BJ2** + **Monocytes** (Scheme Ic); Fibroblasts + Monocytes + IL-4 supplementation, **BJ2** + **Monocytes** + **IL-4** (Scheme Id); Fibroblasts + Macrophages, **BJ2** + **Macrophages** (Scheme Ie); Fibroblasts + Macrophages, **BJ2** + **Macrophages** + **IL-4** (Scheme If).
- *In ECG medium (supplemented with endothelial supplement mix) with or without IL-4 (10 ng mL*^−1^*):* Endothelial cells alone, **HUVECs** (Scheme IIa); Endothelial cells alone + IL-4 supplementation, **HUVECs** + **IL-4** (Scheme IIb); Endothelial cells + Monocytes, **HUVECs** + **Monocytes** (Scheme IIc); Endothelial cells + Monocytes + IL-4 supplementation, **HUVECs** + **Monocytes** + **IL-4** (Scheme IId); Endothelial cells + Macrophages, **HUVECs** + **Macrophages** (Scheme IIe); and Endothelial cells + Macrophages, **HUVECs** + **Macrophages** + **IL-4** (Scheme IIf).
- *In ECG medium (supplemented with endothelial supplement mix* + *10% FBS):* Fibroblasts + Endothelial cells, **BJ2** + **HUVECs** (Scheme IIIa); Fibroblasts + Endothelial cells + Monocytes, **BJ2** + **HUVECs** + **Monocytes** (Scheme IIIb); Fibroblasts + Endothelial cells + Monocytes, **BJ2** + **HUVECs** + **Macrophages** (Scheme IIIc).

#### Metabolic activity

To assess metabolic activity, samples were incubated with 10% v/v Resazurin (Fluorometric cell viability kit I, PromoKine, Germany) in cell culture medium for 2 h. The substrate becomes fluorescent (red) when incubated with viable cells due to reduction. The amount of fluorescence was monitored with a SAFAS Xenius XML fluorescence reader (SAFAS, Monaco) at excitation wavelength of 560 nm and emission wavelength of 590 nm [in AU (Arbitrary Unit)].

#### Cytokine detection by ELISA

Cell culture media were collected at days 1, 3, 6, 10, and 13 and the cytokine amounts in the media were quantified by ELISA developer kits. Absorbance measurements were done at 450 nm. The cytokine amounts were calculated using the standard curves. Optical density (OD) cutoff is set as the OD value for standard concentration of 0 ng mL^−1^. The following cytokines were quantified: transforming growth factor beta (TGF-β), activin, hepatocyte growth factor (HGF), platelet-derived growth factor (PDGF), angiopoietin 1, IL-4, IL-6, IL-8, IL-1β, TNF-α, IL-1RA. The references for all ELISA tests performed are presented here: TGF-β (R&D Systems DY240); activin (R&D Systems DY338); HGF (R&D Systems DY294); PDGF (PeproTech 900-K04); angiopoietin 1 (R&D Systems DY923); IL-6 (PeproTech 900 K-16); IL-8 (R&D Systems DY208); IL-1β (Capture: Antobody Solutions AS56-P, Standard protein: PeproTech 200-01B, Detection-Antibody Solutions AS57-B); TNF-α (R&D Systems DY394); and IL-1RA (PeproTech 900-K474).

The cytokines were quantified as a function of the cell culture condition, as shown in Table 1.

**Table 1:** Quantification of cytokines by ELISA tests as a function of the cell culture condition.

**Conditions**	**Quantified cytokines**	
BJ2 +/- IL-4 BJ2 + Monocytes +/- IL-4 BJ2 + Macrophages +/- IL-4	TGF-β Activin	IL-1β TNF- α IL-1RA
HUVECs + IL-4 HUVECs + Macrophage +/- IL-4 HUVECs + Monocytes +/-IL-4	HGF IL-6 PDGF	IL-1β TNF- α IL-1RA
HUVECs + BJ2 HUVECs + BJ2 + Monocytes HUVEC + BJ2 + Macrophages	HGF Activin Angiopoietin 1 IL-6 IL-8	IL-1β TNF-α IL-1RA

#### Cell pre-labeling with calcein

For co-culture experiments fibroblasts, HUVECs, and THP-1 cells were pre-labeled with calcein before the encapsulation in gelatin. Fibroblasts and HUVECs were stained with green calcein (Vybrant Cell Adhesion Assay Kit (V-13181), ThermoFisher Scientific), whereas THP-1 cells were stained with red-orange calcein [CellTrace™ Calcein Red-Orange, AM (C34851) ThermoFisher Scientific].

The fluorescent indicators, green and red calcein, were prepared as 1 mM stock in DMSO and stored at −20°C until use. Cells were centrifuged to get cell pellet and were resuspended in staining solution. Cells were labeled at a concentration of 1 × 10^6^ cells mL^−1^ and a dye concentration of 5 μM (in serum free medium) of calcein for 30 min at 37°C in the dark and subsequently washed twice in PBS. Cells were resuspended in gelatin for encapsulation.

#### Cell labeling with vimentin/phalloïdin/PECAM-1/DAPI

Before labeling, encapsulated cells were fixed with a 3.7% (v/v) solution of paraformaldehyde (PFA) in PBS for 15 min and then washed three times with PBS.

For morphological characterization of HUVECs, cells were then incubated in a Triton X solution (0.1% in PBS) for 5 min. Then two rinsing steps with PBS were performed, and the samples were incubated for 20 min with bovine serum albumin (BSA) solution (1% v/v) in PBS. After incubation with Triton X and BSA solution, samples were incubated for 90 min with primary antibody (0.938 mg mL^−1^, mAb mouse anti-human, Thermo Scientific) against PECAM-1 (CD31, cell-cell contact related cell membrane protein) at a dilution of 1/150 in PBS. Then the samples were incubated for 30 min with secondary antibody (2 mg mL^−1^ Oregon green 488 conjugate, goat anti-mouse IgG, Thermo Scientific) at a dilution of 1/200 in PBS and two rinsing steps with PBS were performed.

F-actin filaments for fibroblasts, HUVECs, and THP-1 cells were labeled with phalloïdin. The samples were incubated for 1 h with phalloïdin (6.6 μM Alexa Fluor 568 phalloïdin, Molecular Probes Life Technologies) at a dilution of 1/40 in BSA solution (1% v/v in PBS) and two rinsing steps in PBS were performed.

After treatment with a blocking solution (10% goat serum) for 20 min, fibroblast cells were stained with primary anti-vimentin (Vimentin V9, mouse IgG1, Santa Cruz) for 1 h at a dilution of 1/100 in 5% goat serum solution. The samples were rinsed three times for 5 min with PBS and treated with the appropriate fluorophore-conjugated secondary antibodies (goat anti-mouse, Oregon Green) for 1 h at a dilution of 1/200 in 5% goat serum solution.

Finally, at the end of each staining, the samples were incubated for 5 min in DAPI (1 mg mL^−1^, Promokine) at a dilution of 1/100 in PBS and two rinsing steps were performed.

#### Microscopy characterization

Fluorescence images were captured using Nikon Eclipse Ti-S with a 10 × PL Fluor (0.30 NA) objective equipped with Nikon Digital Camera (with NIS-Elements software) and processed with ImageJ. For 3-D images, the labeled cells were analyzed with confocal laser scanning microscope (Zeiss LSM 710, Germany). Excitation/emission wavelength for green calcein was 489/556 nm, 577/599 nm for red calcein, 578/600 nm for Alexa Fluor® 568 Phalloïdin, 489/556 nm for vimentin, and 358/461 nm for DAPI.

For scanning electron microscopy (SEM), the samples were fixed with 4% glutaraldehyde. The specimens were washed with DPBS, prior to a dehydration protocol using an alcohol series of increasing concentrations (70, 95, and 2 × 100%), with incubation periods of 5 min each. Subsequently, samples were incubated in 100% ethanol/hexamethyldisilazane (HMDS) (1:1) for 5 min, then only in HMDS for 2 × 5 min and were dried overnight. Samples were made to adhere onto titanium discs using a carbon tape and were coated with gold/palladium in a sputter coater. The samples were sputtered at 7.5 mA for 3 min under argon atmosphere. Analysis with SEM was performed with a Quanta 400 ESEM (FEI Company, Eindhoven, the Netherlands) at an accelerating voltage of 10 kV.

#### Histology

Before histological analysis, encapsulated cells were fixed with a 3.7% (v/v) solution of PFA in PBS for 15 min and then washed three times with PBS. Then, samples were embedded in paraffin and cut into sections of 4–5 μM and stained with hematoxylin and eosin (H&E). Slides were scanned with Hamamatsu Nanozoomer 2.0 HT Scanner.

#### Statistical analysis

The statistical significance of the obtained data was assessed using the *t-*test, Kruskal–Wallis, Shapiro–Wilk, or Mann–Whitney tests (*n* ≥ 3). The error bars were representative of standard deviation (SD). Differences at *p* ≥ 0.05 were considered statistically insignificant.

## Results and discussion

### Co-encapsulation of fibroblasts with macrophages or monocytes (co-culture system)

**Figure 1:**
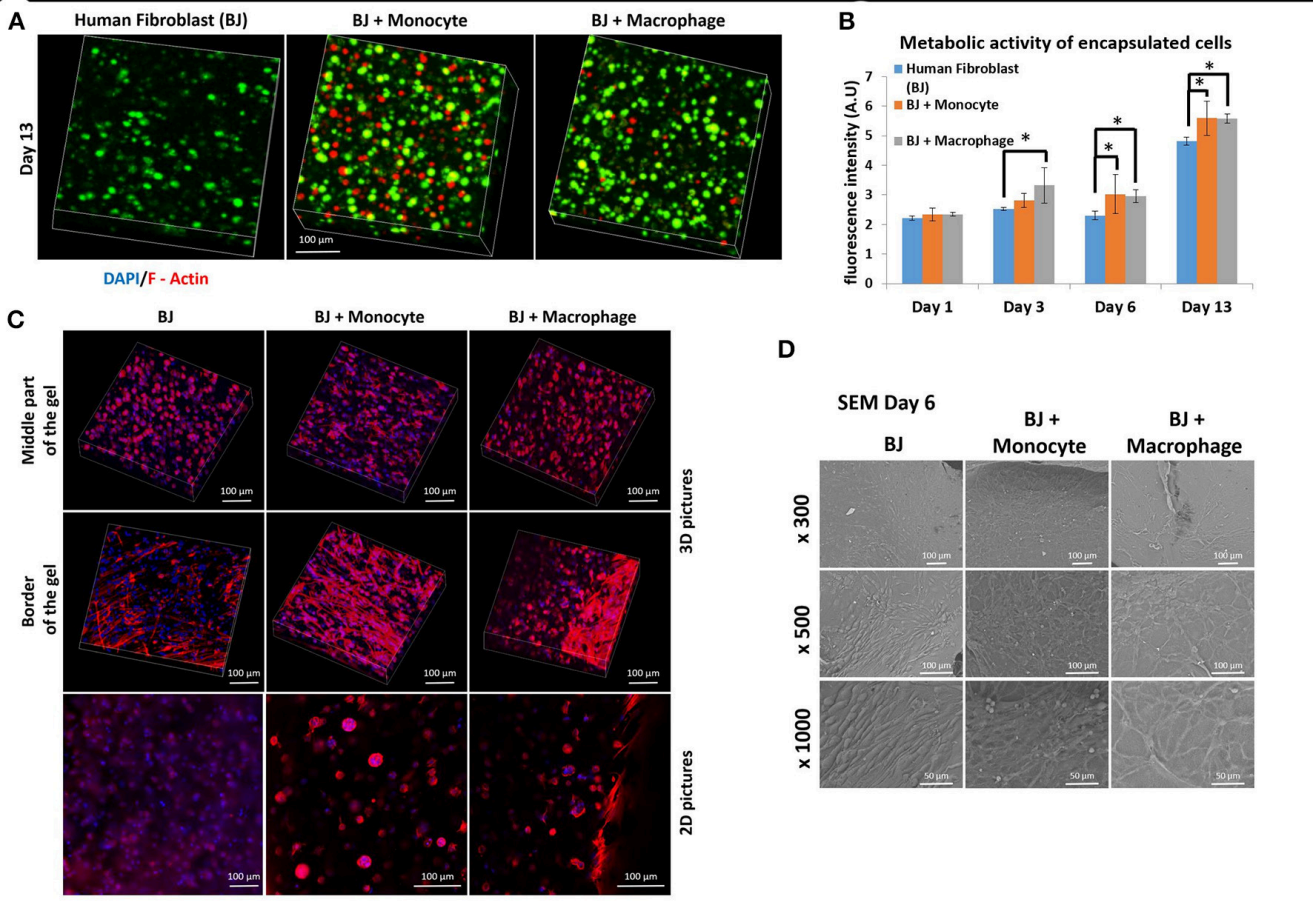
Co-encapsulation of Human fibroblasts (BJ2) in gelatin hydrogel in co-culture condition with either monocyte or macrophage without IL-4 supplementation. **(A)** Confocal pictures of encapsulated cells labeled with green calcein (BJ2) or red calcein (monocyte or macrophage) after 13 days of experiment. **(B)** Follow up of metabolic activity of the different co-culture systems for 13 days (*n* = 3) (*p < 0.05). **(C)** Confocal pictures of DAPI/ Phalloidin (F-actin) stainings and **(D)** SEM pictures of the different co-culture systems after 6 days of experiment.

We first studied the co-encapsulation of human fibroblasts (BJ2) with either monocytes (naive THP-1 cells) or macrophages (PMA activated THP-1 cell). The first step was to check if macrophages or monocytes were still persistent in the hydrogel after 13 days of experimentation. Different cell types were distinguished by pre-labeling BJ2 cells with green calcein and monocytes or macrophages with red calcein (Figure 1A) (Ratio of the cells were 6:1 BJ2/THP-1). In Figure 1A, we could observe that macrophages or monocytes were still persistent after 13 days of experimentation. Moreover, the number of remaining immune cells was higher in the case of BJ2/monocyte co-culture condition. This result can be explained by the fact that once activated with PMA macrophages can no longer proliferate. The presence of macrophages and monocytes had a boosting effect on the metabolic activity of the overall system (Figure 1B). At day 1, the metabolic activity was the same among all conditions (BJ2, BJ2 + Monocyte, and BJ2 + Macrophage), but after 3 days the increase in the metabolic activity became significant for BJ2 + Macrophage and after 6 days for BJ2 + Monocyte. These results indicate that the boosting effect on the proliferation cannot be attributed to the higher number of cells present in the beginning of the experiment alone.

Cellular organization in the hydrogel was also checked by performing DAPI/F-Actin staining (Figure 1C). With confocal 3-D reconstructions, when we looked at the center of the hydrogel, we could see a different level of cellular organization between BJ2 alone and BJ2 + Macrophage or BJ2 + Monocyte. In the case of co-culture condition, we observed more spreading of the cells, whereas in BJ2 alone condition cells assumed a more spherical morphology. At the border of the gel, areas with extensive cellular organization were observed with co-culture conditions, especially in the condition with monocyte, indicating denser structure with well extended F-Actin filaments. This might indicate that the presence of macrophages enables faster outward movement of the cells from the hydrogels. In co-culture conditions, the presence of monocytes or macrophages resulted in the formation of clusters, which was not seen in conditions where BJ2 cells were encapsulated alone in the hydrogel.

In order to better characterize the interactions of cells with the surrounding hydrogel, SEM pictures were taken after 6 days of experimentation (Figure 1D). Extensive cellular coverage of cells with lamellipodia was apparent on both macrophage and monocyte systems, whereas in monocyte condition individual spherical monocytes were also seen. Moreover, in both monocyte and macrophage systems, zones with fibrillary components resembling ECM secretion were also observed on the SEM pictures. In the case of monocytes, spherical monocytes in contact with fibroblasts were clearly observed. The co-culture conditions increased cell-cell contact and resulted in denser web-like cellular clusters within and on top of the hydrogels.

**Figure 2:**
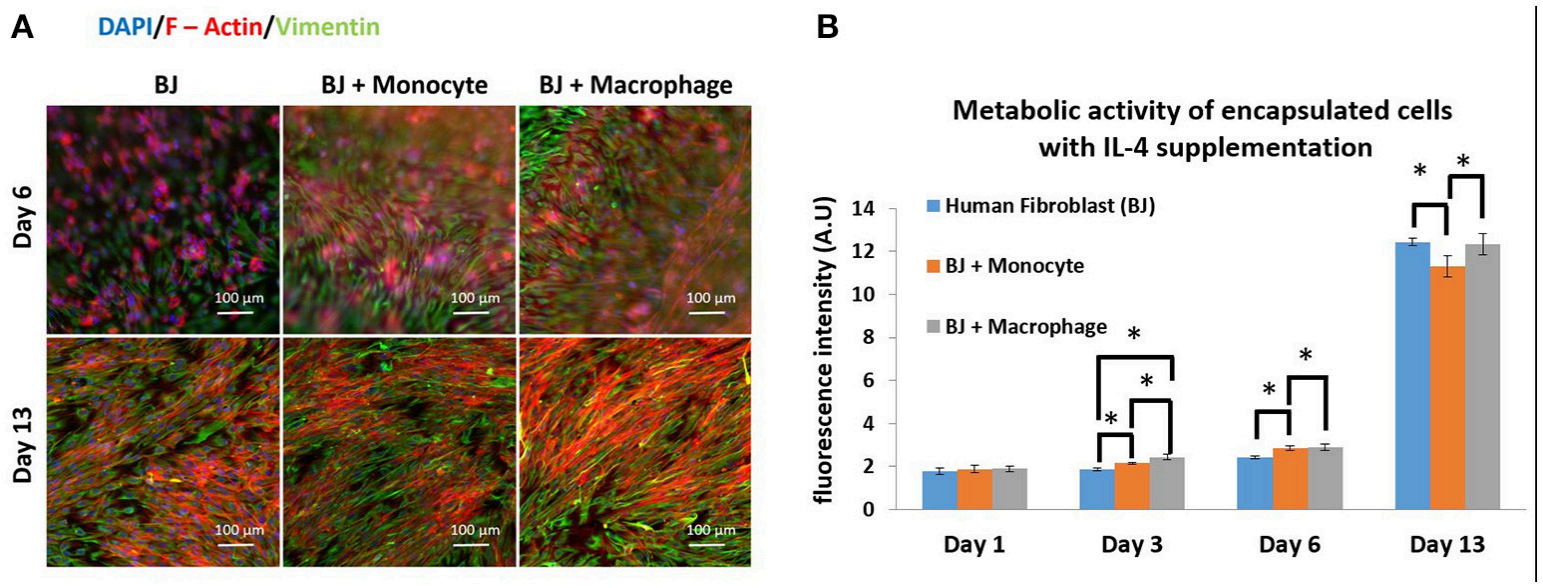
Co-encapsulation of Human fibroblasts (BJ2) in gelatin hydrogel in co-culture condition with either monocyte or macrophage with IL-4 supplementation. **(A)** Pictures obtained with epifluorescence microscope of encapsulated cells stained with DAPI/Phalloidin (red)/Vimentin (green). **(B)** Follow up of metabolic activity of the different co-culture systems for 13 days (*n* = 3) (*p < 0.05).

The next step was to investigate the effect of IL-4 supplementation in the supernatant on the overall co-culture system. Supernatant was supplemented with 10 ng mL^−1^ IL-4 each time the medium was changed (day 3, 7, and 10). As previously explained, IL-4 was chosen to induce resident macrophage like phenotype and to promote tissue remodeling and inflammation resolution. For all the conditions, IL-4 supplementation seemed to have a boosting effect on the metabolic activity of the overall system. Without IL-4, the metabolic activity reached values around 6 AU, and with IL-4, we reached values around 12–13 AU (Figure 2B). The presence of macrophages had a significant effect on metabolic activity until day 6, whereas on day 13 in BJ2 alone condition with IL-4 supplementation the metabolic activity was significantly high when compared to no IL-4 conditions. This means that IL-4 has particularly induced fibroblast proliferation even without immune cells. Vimentin is an intermediate filament protein with important roles in cell integrity, migration, and stability. It has been shown that vimentin deficiency decreases the fibroblast's ability to migrate (Eckes et al., 1998). Moreover, it has been shown that during monocyte to macrophage differentiation there is an increase in vimentin expression (Benes et al., 2006). In order to observe the potential effect of IL-4 and monocyte/macrophage presence on vimentin expression, the system was immunostained with vimentin (Figure 2A). On day 6, a denser cellular body was apparent with both monocyte and macrophage containing conditions, where the staining is stronger for macrophage samples. By day 13, the difference in staining between monocytes and macrophages was less obvious with more overlap in actin filaments in the case of macrophage containing structures. High level of actin/vimentin co-localization has been observed with macrophages previously consistent with our observations (Correia et al., 1999). Moreover, the addition of IL-4 did not have an effect on the clusters (Supplementary Figure 1). With or without IL-4 supplementation, we can always observe the formation of clusters for co-culture conditions of BJ2 with monocyte or macrophage.

**Figure 3:**
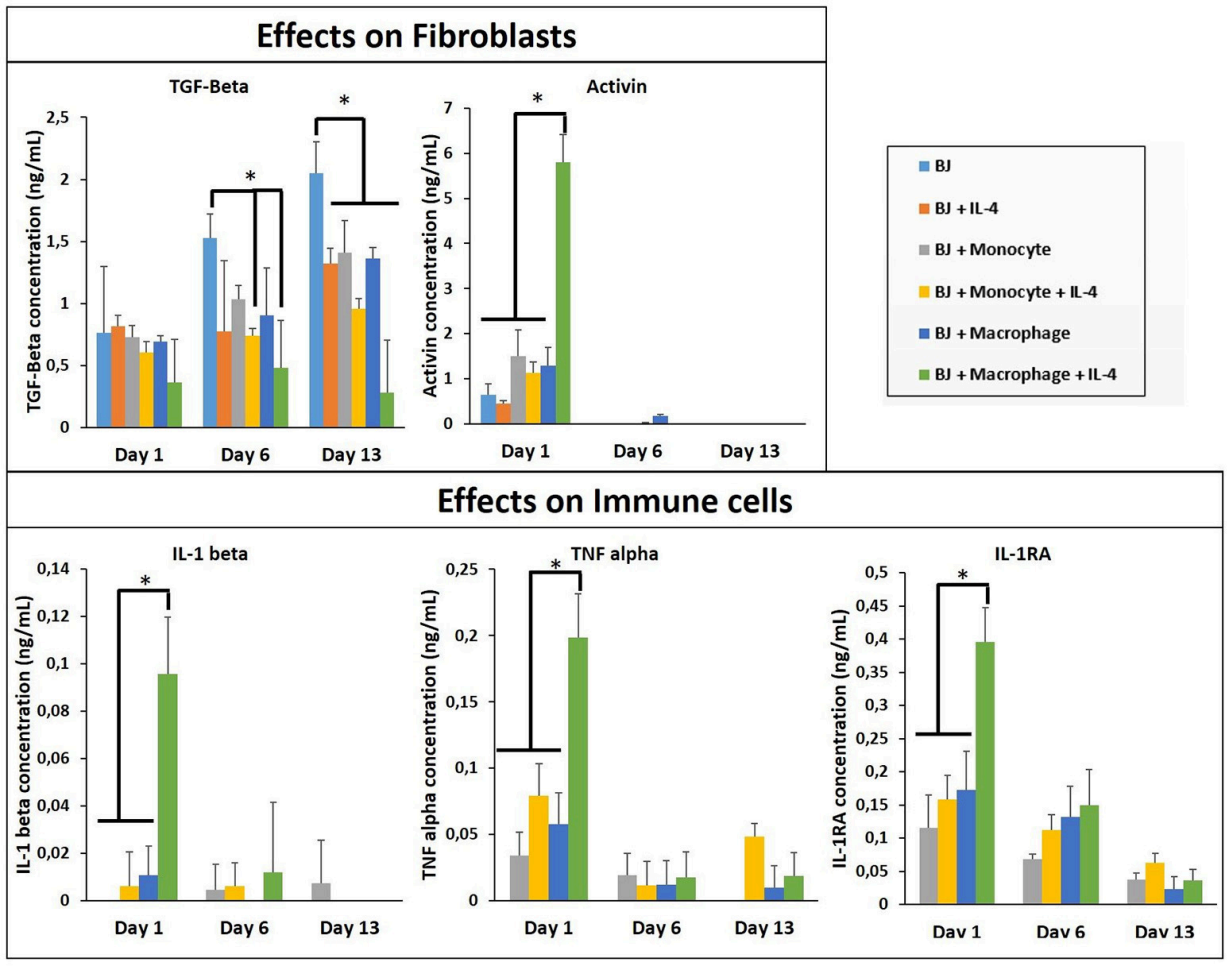
Cytokine quantification (TGF-β, Activin, IL-1β, 1I-1R1, and TNF-α) in the supernatant at different time point for the co-culture experiments with and without IL-4 supplementation (*n* = 3) (*p < 0.05).

In all co-culture conditions, cytokine/growth factor analyses were done with three goals: (i) detection of the effect of created microenvironment on the secretion of cytokines/growth factors related to the function of the non-immune component, (ii) the presence and the stability of the supplemented cytokine (IL-4), and (iii) the effect of created microenvironment on the secretion of pro-/anti-inflammatory cytokines. As shown in Figure 3, the stability of the supplemented IL-4 was demonstrated with its quantification in the supernatant at different time points during the cell culture. The possible secretion of IL-4 from the different cell types could not be evaluated since this interleukin was also supplemented in the medium. In the case of fibroblasts, the most important effect is a significant boost of activin, IL1-β, TNF-α, and IL-1RA secretion at day 1 in the presence of activated macrophages and IL-4 (Figure 3). The presence of IL-4 resulted in an increase in secretion creating a cytokine enriched environment, regardless of the pro- or anti-inflammatory nature of the cytokines. Activin is a potent growth factor involved in proliferation of fibroblasts; the initial boost in activin expression at day 1 time point might also be related to the significant increase in IL-1β and TNF-α presence, which have been previously shown to induce activin secretion (Hübner and Werner, 1996). IL-1β and TNF-α are pro-inflammatory cytokines secreted by M1 macrophages, whereas IL-1 receptor antagonist is a M2 marker for cytokine stimulated macrophage polarization. However, the presence of 3-D proteinaceous microenvironment available to monocytes and macrophages under encapsulation conditions creates intermediate phenotypes that would be difficult to define in M1/M2 paradigm. Previously, in animal studies, it had been shown that the overexpression of IL-1β and TNF-α significantly increased collagen deposition and also fibronectin expression (Margetts et al., 2002). Prolonged expression of pro-inflammatory markers might have pathological outcomes (such as fibrosis), but an initial boost in cytokines in the context of *in vitro* tissue maturation can be highly beneficial for faster proliferation, ECM secretion, and organization of the cells within the artificial tissue.

The cytokine boosting effect in the presence of IL-4 and macrophage was seen with all the conditions, except TGF-β. For the three conditions (BJ2, BJ2 + Monocytes, and BJ2 + Macrophages) the production of TGF-β increases with cell culture time (from day 1 to 13). Previously a similar effect was demonstrated by Dardalhon et al. (2008) where the presence of IL-4 decreased TGF-β expression in T cells, which in turn resulted in inhibition of TGF-β induced T-reg cell differentiation (Dardalhon et al., 2008). In co-encapsulation conditions, the presence of IL-4 in medium has an important effect on downregulating the TGF-β production at day 6 for both conditions. This downregulation becomes significant at day 13 for all co-encapsulation conditions, even without IL-4 after 13 days of experimentation, so the presence of monocyte or macrophages seems to have an effect on TGF-β secretion.

### Co-encapsulation of endothelial cells/macrophages or monocytes (co-culture system)

**Figure 4:**
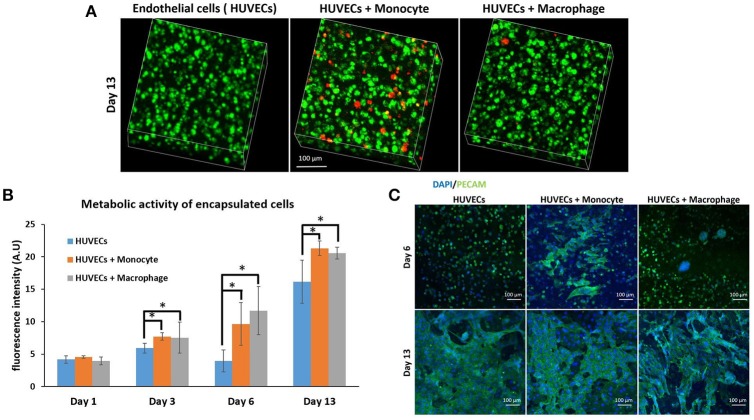
Co-encapsulation of endothelial cells (HUVECs) in gelatin hydrogel in co-culture condition with either monocyte or macrophage without IL-4 supplementation. **(A)** Confocal pictures of encapsulated cells labeled with calcein green (HUVECs) or calcein red (monocyte or macrophage) after 13 days of experiment. **(B)** Follow up of metabolic activity of the different co-culture systems for 13 days (*n* = 3) (*p < 0.05). **(C)** Confocal pictures of DAPI/ PECAM stainings after 6 and 13 days of experiment.

After the investigation of the effect of co-encapsulation of fibroblasts with immune cells (macrophages or monocytes), we have continued the monocyte/macrophage co-encapsulation with another cell type critical for tissue engineering and regenerative medicine, that is, vascular endothelial cells. For tissue regeneration, obtaining a mature vascular network is crucial to provide nutrient and evacuate waste, and we hypothesize that the presence of macrophages can aid in the formation of capillary networks in hydrogels, and thus the interaction of endothelial cells and immune cells in a co-encapsulation system has been studied. We have used the same protocol as previously described with fibroblasts. Cells were encapsulated in a gelatin hydrogel (6% w/v) at a density of six million HUVECs and one million monocytes or macrophages per mL of hydrogel. We have also checked if macrophages or monocytes were still persistent after 13 days of co-encapsulation experiment with HUVECs by using the same strategy to pre-label the cells with green calcein (HUVECs) and red calcein (macrophages or monocytes). In Figure 4A, we have shown that macrophages or monocytes were still persistent after 13 days of experimentation, with higher number of remaining monocytes when compared to macrophages for the same reasons explained previously (once activated, macrophages can no longer proliferate).

The metabolic activity of the system was also followed for 13 days (Figure 4B) and the effect was similar to the case of fibroblasts. The presence of macrophages and monocytes in the co-encapsulation system with HUVECs had a boosting effect on the metabolic activity of the overall system, which cannot be attributed to the higher number of cells at the beginning of the experiment alone since the metabolic activity of all conditions were equivalent at day 1. Our primary findings have shown that the addition of macrophage or monocyte in a co-encapsulation system with connective tissue cells such as fibroblasts or endothelial cells has a boosting effect on the proliferation of these cells. The effect cannot be attributed only to the proliferation of monocytes either, as there was no significant difference between the conditions with monocyte and macrophage (non-proliferative) co-encapsulation; both conditions had higher metabolic activity when compared to HUVECs alone condition.

We have performed DAPI/phalloïdin (F-actin) staining and observed the formation of large clusters after 6 days of experimentation for the condition HUVECs + Macrophages (Supplementary Figure 2). These clusters were still present after 13 days of experimentation. For the condition HUVECs + Monocytes, these clusters were much smaller and easier to observe after 13 days of culture. So there is a correlation between HUVECs and fibroblasts when co-encapsulated with macrophages or monocytes, which can be attributed to the behavior of monocytes/macrophages within the hydrogel. In both cases, we can see the formation of clusters which cannot be seen when fibroblasts or HUVECs are encapsulated alone in the hydrogel.

PECAM CD31 staining was also performed after 6 and 13 days of experimentation (Figure 4C) to check endothelial cell organization within the hydrogel. This a membrane protein found on the surface of an endothelial cell, which is a marker of endothelial intercellular junctions. It is a well-known endothelial marker that establishes at cell-cell junctions (Ford et al., 2006). PECAM is expressed at the earliest stage of angiogenesis and this is why its expression is relevant in tissue engineering (Pinter et al., 1999). After 6 days of culture, there was limited organization in the case of HUVECs alone and co-culture HUVECs + Macrophages, except for the presence of large clusters in the case of co-culture. For HUVECs + Monocytes condition after 6 days of experimentation, we could see increased PECAM staining indicating that the cells were more interconnected. After 13 days of culture, for all conditions, CD31 marker was well expressed and the cells were interconnected. With this experiment, we could conclude that the presence of monocyte seemed to have a positive effect on HUVEC organization at the early stage, since HUVECs expressed more cell-cell connections.

**Figure 5:**
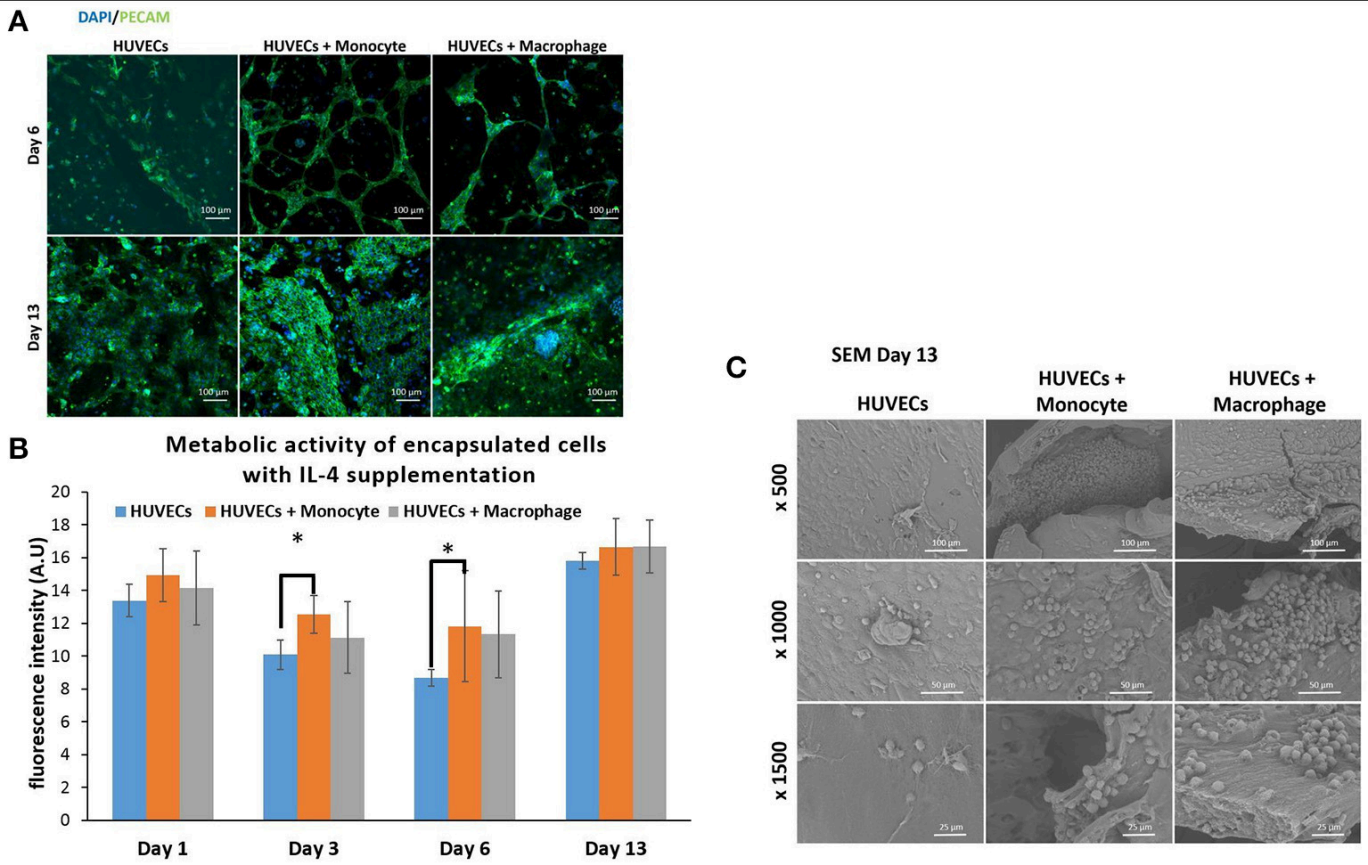
Co-encapsulation of endothelial cells (HUVECs) in gelatin hydrogel in co-culture condition with either monocyte or macrophage with IL-4 supplementation. **(A)** Confocal pictures of DAPI/ PECAM stainings after 6 and 13 days of experiment. **(B)** Follow up of metabolic activity of the different co-culture systems for 13 days (*n* = 3) (*p < 0,05). **(C)** SEM pictures of the different co-culture systems after 13 days of experiment.

We have also investigated the effect of IL-4 supplementation in the supernatant on the overall co-culture system. Supernatant was supplemented with 10 ng mL^−1^ IL-4 each time the medium was changed. As presented in Figure 5B, IL-4 supplementation had a boosting effect on the metabolic activity of the overall system for all conditions, especially for the first day. Indeed, without IL-4 the metabolic activity reached values around 4–5 AU for all conditions on the first day, whereas with IL-4 the metabolic activity reached values around 15 AU. After that we could notice a small decrease at days 3 and 6, and then the metabolic activity increased again to reach values around 16–17 AU for all conditions. The analysis of metabolic activity of the overall co-encapsulation system with endothelial cells have shown that IL-4 induced the proliferation of endothelial cells at the early stage, but then the proliferation seemed to stop and most probably in the benefit of cell organization in this 3-D microenvironment.

Moreover, DAPI/phalloïdin staining performed at days 6 and 13 has also shown that the addition of IL-4 did not have an effect in the presence of clusters (Supplementary Figure 3). After 6 days, we were able to see larger clusters in the case of HUVECs + Macrophages when compared to HUVECs + Monocytes. Over time, it seemed that the clusters became even bigger for these two conditions.

PECAM CD31 staining was also performed to check the cellular organization in this hydrogel (Figure 5A). After 6 days of experimentation, we could see more organization for HUVECs alone in the hydrogel when compared to HUVEC alone samples without IL-4. For the two co-encapsulation conditions (HUVECs + Monocytes and HUVECs + Macrophages), the effect was more striking since we could observe that the HUVECs started to organize in a vessel-like structure with more sprouting. In the presence of monocytes/macrophages, the macroscopic organization resembled that of sprouting capillaries on day 6. After 13 days, most of the cells were interconnected and we could observe almost the same organization for all the conditions except for HUVECs + Macrophages where some tubular structures could be seen.

With this experiment we have seen that even though IL-4 did not affect the presence of clusters, it seemed to have an effect on their size over time by promoting their growth. Moreover, in the presence of IL-4, we could see faster organization of HUVECs and higher intercellular junctions especially on the first day of the experiment. The positive effect of macrophages on angiogenesis have already been demonstrated with capillary sprouting assay using beads (Tattersall et al., 2016). In this study, it was shown that IL-4 stimulation resulted in less developed sprouts, but in our case, IL-4 stimulation triggered the sprouting of vessel in the earliest stage. The difference can be attributed to the change in cell microenvironment (3-D cell culture in degradable hydrogel) together with the proliferation boosting effect of IL-4. In the earliest stage, the proliferation of HUVECs under IL-4 stimulation was higher and as a consequence they were able to easily remodel the hydrogel to organize themselves into a vessel like structure.

In order to see the cell-cell interactions between monocytes/macrophages with HUVECs at higher magnifications, we carried out SEM. In the case of HUVECs only condition, connected layers of well-spread endothelial cells were visible with occasional cellular clusters (Figure 5C), and with the addition of monocytes and macrophages, the two cell populations could be distinguished as the well-spread endothelial cells interacting with spherical monocytes/macrophages (Figure 5C). The clusters which were also observed in confocal microscopy images seemed to be mostly formed from the immune component (Figure 5C).

**Figure 6:**
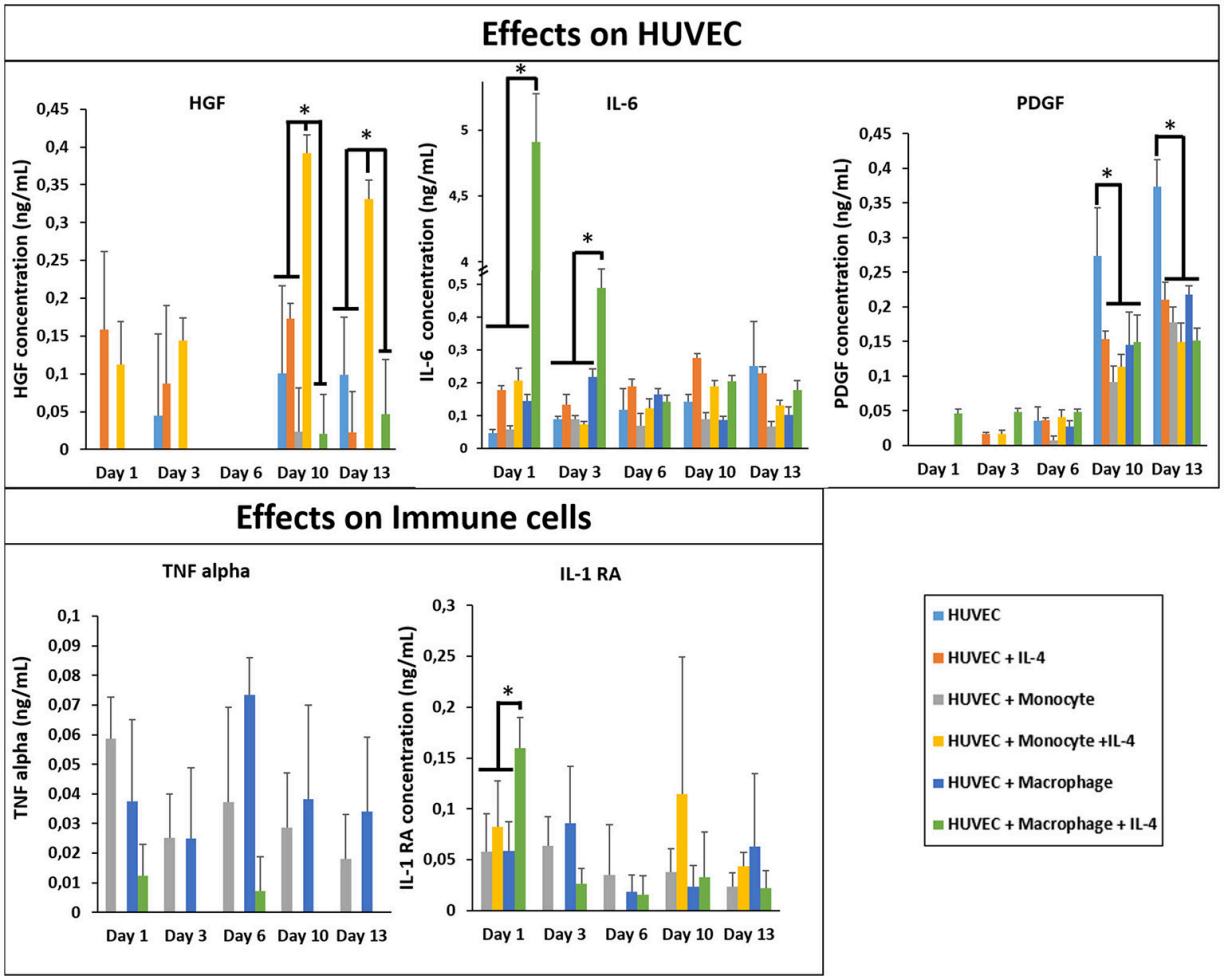
Cytokine quantification (HGF, PDGF, IL-1RA, IL-6, and TNF-α) in the supernatant at different time point for the co-culture experiments with and without IL-4 supplementation (*n* = 3) (*p < 0.05).

In Figure 6, we first checked and demonstrated the stability of the supplemented IL-4 with its quantification in the supernatant at different time point during the cell culture. The possible secretion of IL-4 from the different cell types could not be evaluated since this IL was also supplemented in the medium. The presence of IL-4 resulted in a production boost of IL-6 and IL-1RA in the presence of macrophages in a similar manner as in the case of fibroblasts (Figure 6). However, in the presence of HUVECs the boosting effect was not observed for TNF-α, showing that the cytokine release profiles were culture condition dependent and could not be explained based on the effect of IL-4 and encapsulation conditions on monocytes and macrophages. In a similar vein, the upregulation of HGF was the most prominent for the case of HUVEC + Monocyte condition after 10 days of culture, with the difference being significant after day 10 and day 13. Hepatocyte growth factor is a potent growth factor active in angiogenesis and shown to be mostly effective on endothelial and epithelial cells. Previously, HGF has been shown to induce a regulatory phenotype for monocytes (Rutella et al., 2006), and thus the cross talk between HUVECs and monocytes seems to induce an increase in its secretion. It is possible to see that HGF was also released from HUVEC + Macrophage in presence of IL-4. In the case of PDGF, a growth factor which is implicated in cell proliferation and late stages of angiogenesis, there was a steady increase until day 13 for all culture conditions; but its highest expression was observed in HUVECs only conditions (Figure 6). This can be related to the fact that endothelial cells secrete PDGF for recruitment of pericytes for establishing the stability of the newly formed vessel structures (Saik et al., 2011); in the presence of monocytes and macrophages such a support component is readily available which can result in the downregulation of PDGF secretion, whereas in the case of HUVECs only no such support is forthcoming so the secretion continues steadily as a function of increasing number of endothelial cells. The downregulation of PDGF in the case of IL-4 presence for HUVEC only conditions can be related to its induction by cell-cell contact proteins in HUVECs (Iademarco et al., 1995), which might have a negative feedback effect on recruitment via PDGF thus resulting in a decrease in secretion.

### Co-encapsulation of fibroblasts/endothelial cells/macrophages or monocytes (tri-culture system)

**Figure 7:**
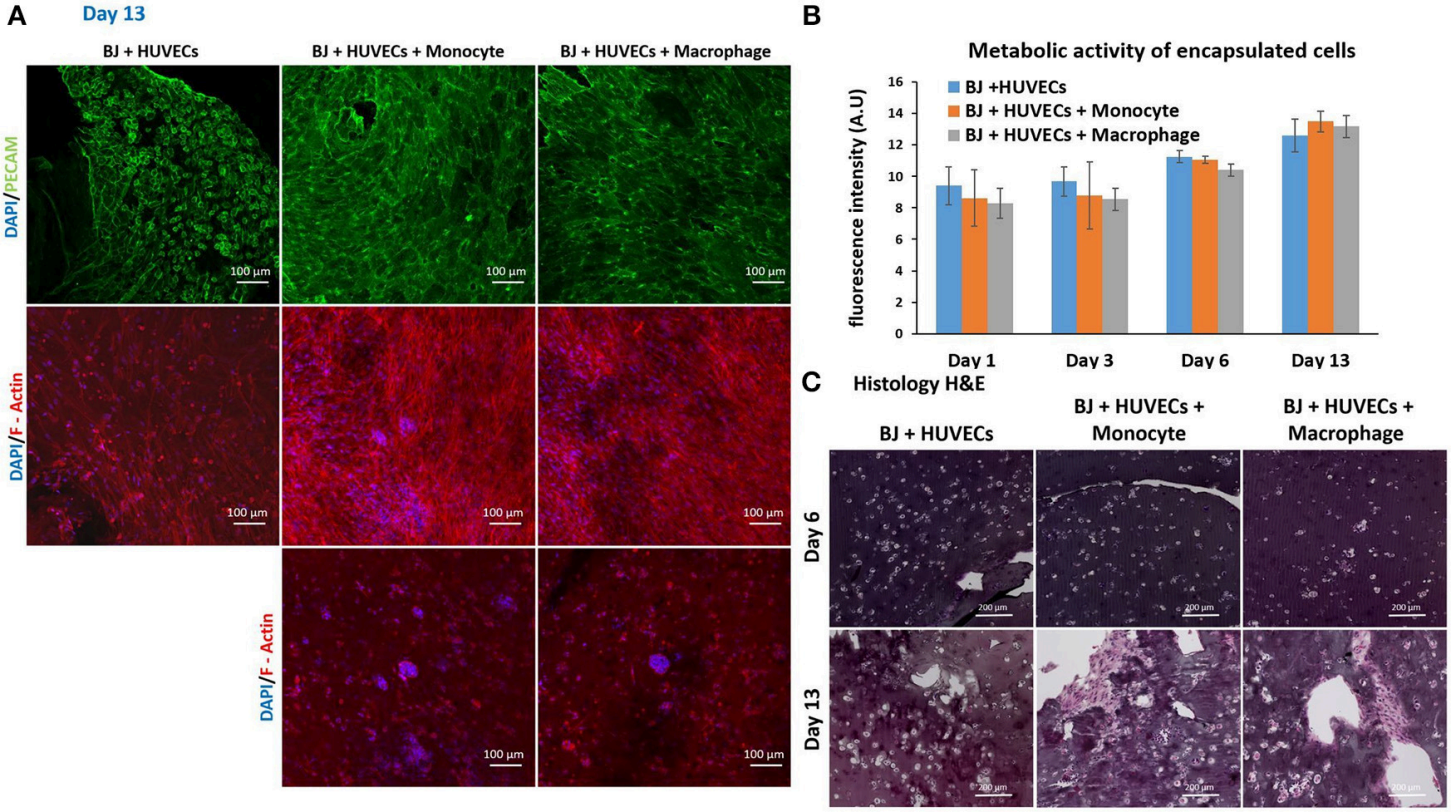
Co-encapsulation of endothelial cells (HUVECs)/ Fibroblast (BJ2) in gelatin hydrogel in tri-culture condition with either monocytes or macrophages without IL-4 supplementation. **(A)** Confocal pictures of DAPI/PECAM/Phalloidin stainings after 13 days of experiment. **(B)** Follow up of metabolic activity of the different tri-culture systems for 13 days (*n* = 3). **(C)** Histology pictures (Hematoxylin/Eosin stainings) of the different co-culture systems after 6 and 13 days of experiment.

Finally, we studied the co-encapsulation of both fibroblasts and endothelial cells with monocytes or macrophages to recreate the *in vivo* microenvironment with immune and connective tissue cells. We checked the metabolic activity of the different systems (Figure 7B). The metabolic activity remained constant and at the same level for all conditions (BJ2 + HUVECs and BJ2 + HUVECs with monocytes or macrophages).

Moreover, the level was close to what we found for co-encapsulation of HUVECs with immune cells with IL-4 supplementation. In this configuration, we promoted cellular organization instead of proliferation without cytokine supplementation. In Figure 7A, we can see well defined intercellular junctions after 13 days of experimentation (PECAM staining), which was denser in the presence of macrophages or monocytes. The staining of F-actin filaments (Figure 7A) confirmed these findings by showing denser tissue-like structure after 13 days of experimentation in the presence of macrophages or monocytes. We could also see the presence of clusters for these two conditions. H&E staining was performed for all conditions to check cellular organization and tissue remodeling (Figure 7C). After 13 days of experimentation, we observed more cellular migration, especially more hydrogel degradation and remodeling in the presence of macrophages or monocytes. We were still able to observe the clusters.

**Figure 8:**
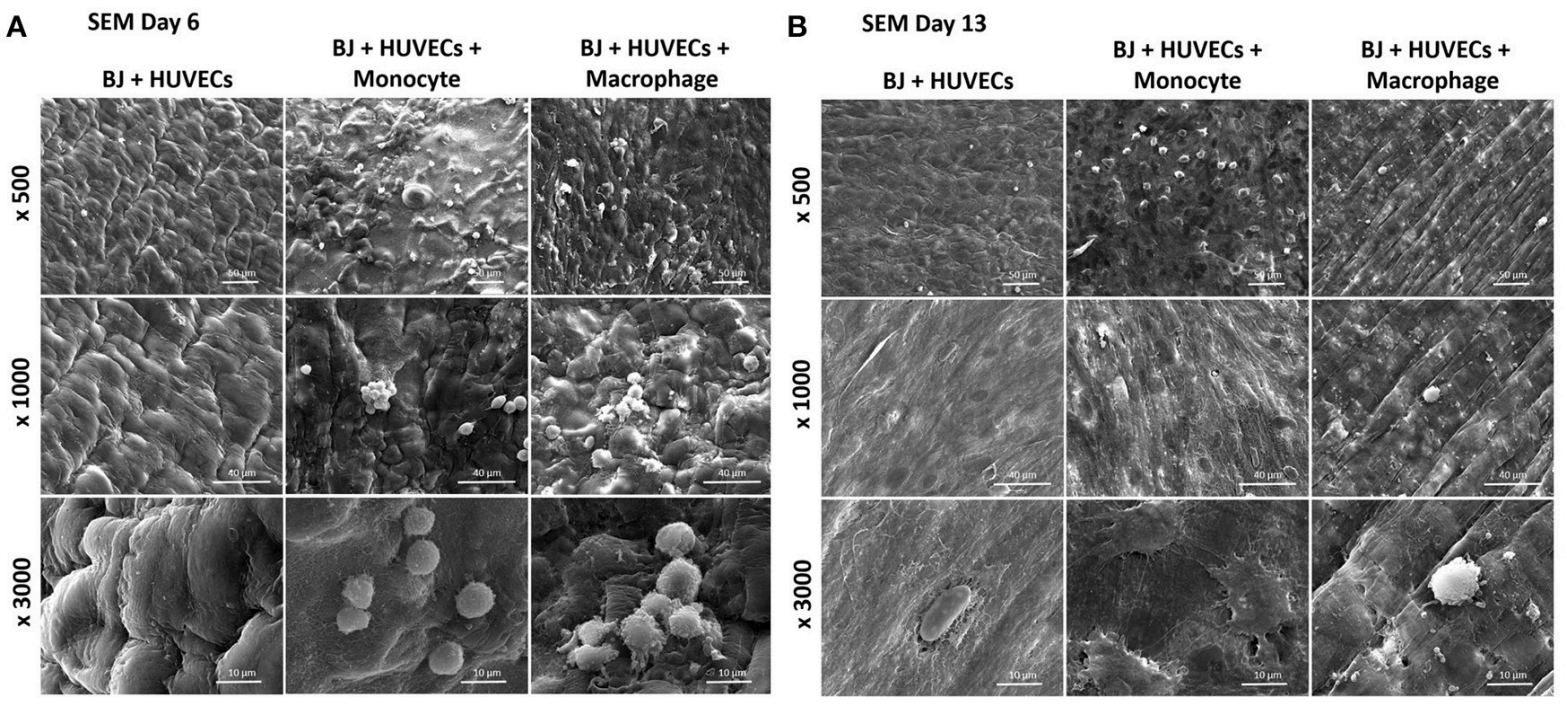
Co-encapsulation of endothelial cells (HUVECs)/Fibroblast (BJ2) in gelatin hydrogel in tri-culture condition with either monocyte or macrophage without IL-4 supplementation. SEM pictures of the different co-culture systems after 6 **(A)** and 13 **(B)** days of experiment.

The co-culture of fibroblasts and HUVECs tri-culture conditions resulted in more remodeled gel structures at higher magnification in SEM compared to other single or co-culture conditions. The fibroblast/HUVEC culture alone by day 6 resulted in more contracted hydrogel structures with pleated surface features by day 6, indicating contraction of the 3-D microenvironment by the activities of the encapsulated cells (Figure 8A). The monocytes and macrophages could be distinguished as separate spherical cells interacting with other cells by day 6. The difference with the previous conditions was more evident by day 13 as the hydrogel substrate has highly organized collagen-like structures, particularly in the case of macrophage containing tri-culture system (Figure 8B). Moreover, unlike previous cases, the clusters of macrophages were less prominent at high magnifications and dense highly remodeled structures were observed (Figure 8B). Together with confocal microscopy images showing dense cellular layers, histology images showing higher hydrogel degradation, and cellular movement and SEM images showing highly organized ECM like structures with cellular components; it can be concluded that the presence of the monocytes and macrophages has a distinct synergistic effect on remodeling and integration of gelatin hydrogel based scaffolds.

**Figure 9:**
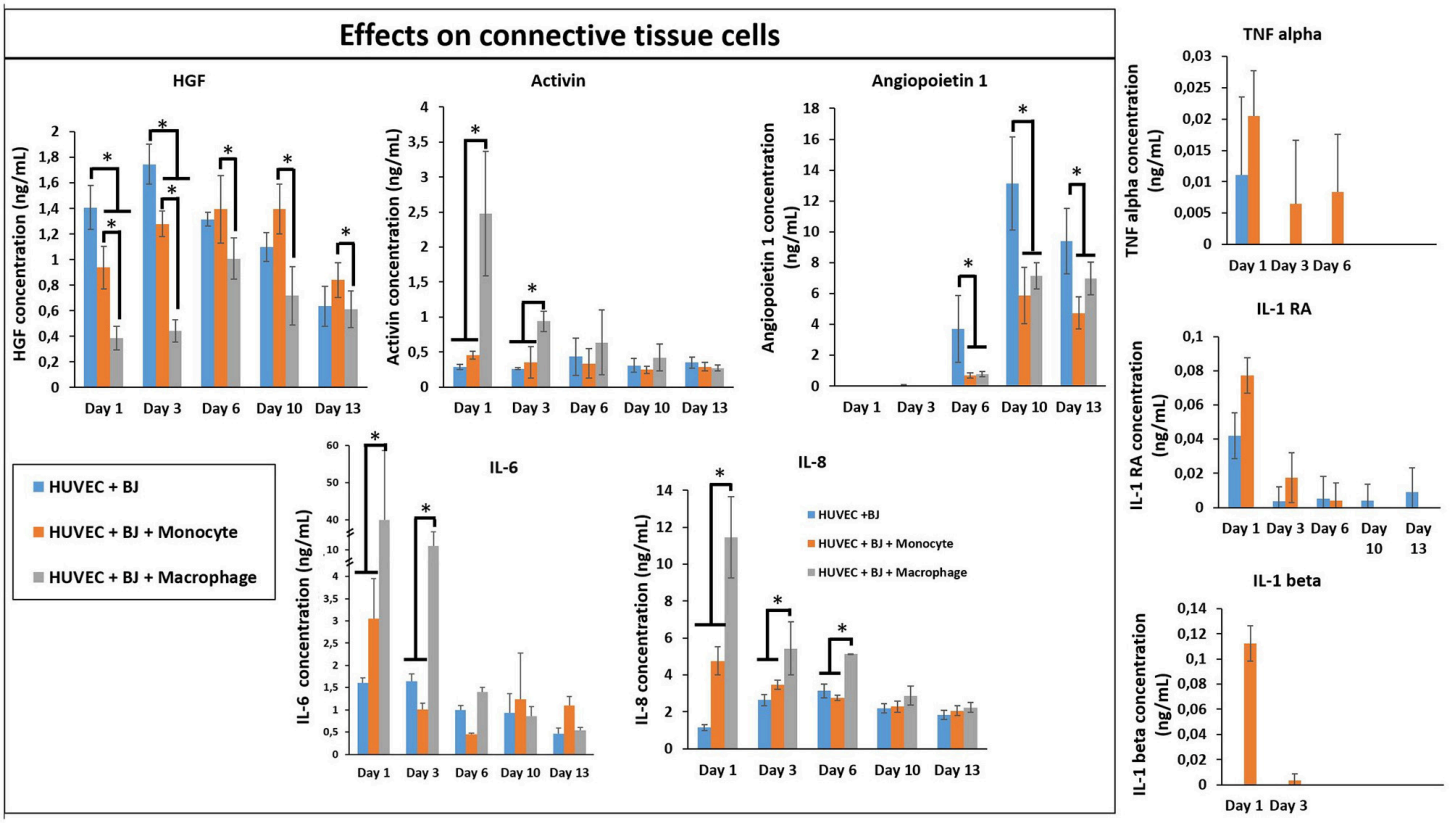
Cytokine quantification (Angiopoetin-1, Activin, HGF, IL-1β, IL-1RA, IL-6, IL-8, and TNF-α) in the supernatant at different time point for the tri-culture experiments (*n* = 3) (*p < 0.05).

In order to see the effect of tri-culture conditions on cytokine secretion, cytokine screening similar to that of co-culture conditions was carried out. The presence of macrophages resulted in a significant boost in the secretion of activin, IL-6, and IL-8, which are important in connective tissue cell proliferation and angiogenesis (Figure 9), previously obtained in a similar manner from co-culture conditions in the presence of IL-4 and macrophages. However, one additional advantage of tri-culture conditions was the longer period of the boost observed; in IL-4/macrophage case the boost was generally limited to the first day of culture, whereas in tri-culture conditions the effect was sustained at a significant level for at least 3 days (6 days for IL-6 and IL-8). Similar to the case of HUVEC/monocyte/macrophage encapsulation conditions (but in the presence of IL-4), HGF secretion was lower in the presence of macrophages. For the case of monocytes, it steadily increased and became significantly higher when compared to macrophage containing tri-culture conditions, suggesting that the presence of the fibroblasts provides additional cues resulting in effects similar to that of biochemical stimulation. Angiopoeitin-1, is one of the ligands of TIE receptors expressed by endothelial cells and macrophages and is highly implicated in angiogenesis (Fagiani and Christofori, 2013). Similar to PDGF, it is more active in the maturation and stability of blood vessels. The secretion of angiopoetin became detectable after day 6 for all conditions and significantly increased at day 10. For both timepoints the secretion was higher for HUVEC/fibroblast co-culture when compared to the monocyte/macrophage containing tri-cultures. This can be related to the presence of more cells with physical support potential in the capillaries formed. The presence of macrophages resulted in higher amount of both pro- and anti-inflammatory cytokine secretion for day 1 (TNF-α, IL-1β, and IL-1RA, respectively), with concentrations similar to that of previous co-culture conditions.

In our model, in the presence of fibroblasts there was a clear increase in the secretion of IL-1β, IL-1RA, and TNF-α between monocytes and differentiated macrophages. When only HUVEC component was present this effect was confounded; however in tri-culture conditions the secretion in the presence of macrophages increased several times, demonstrating the potential effect of macrophages on the biochemical microenvironment.

Previously, interactions between three cell types have been studied in other contexts, with synergistic and antagonistic effects. For example, a hydrogel-based 3-D culture system has been used by several groups in order to allow live cell retrieval to investigate the interaction between different cell types under co- or tri-culture configurations. One example is the research done by Rinker et al. (2014), in which the interactions between mesenchymal stem cells, adipocytes, and osteoblasts were studied in a 3-D tri-culture model of hyperglycemic conditions in the bone marrow microenvironment (Rinker et al., 2014). Another example of tri-culture in a hydrogel encapsulation system is the recent work described by Bray et al. (2017), in which a 3-D tri-culture model was developed for the *ex vivo* study of acute myeloid leukemia (Bray et al., 2017). As a hydrogel, they used the matrix metalloproteinase-sensitive hydrogels prepared from PEG and heparin, functionalized with adhesion ligands and pro-angiogenic factors, allowing for the analysis of acute myeloid leukemia development and response to treatment. Furthermore, endothelial cells and mesenchymal stromal cells were co-seeded to mimic the vascular niche for acute myeloid leukemia cells. However, these studies were more related to *in vitro* disease models and not for harnessing its effects for potential therapeutic applications. In this work, we have chosen gelatin as an ECM based hydrogel that can be used in implantable configurations to encapsulate the co- and tri-culture system to better understand the signals of inflammation into the engineered tissues in the form of different cell types (HUVEC, fibroblasts, and macrophages) co-encapsulated in hydrogels with the presence of IL-4.

For the maturation of engineered tissue, cell-cell interactions, growth factors, and morphogens have been widely utilized. However, these maturations are generally done in the form of single culture systems without involving all the cell types present in a given tissue because of the difficulties in establishing such cultures while ensuring the viability and phenotypic stability of all cell types involved. One potential way of circumventing this problem would be to establish a specific microenvironment in the co-culture environment that would mimic a given event during the timeline of healing, so that the cellular interactions can make up for the missing components in the culture medium. In this study, we tried to use the inflammatory components both differentiated macrophages (to mimic the first group of immune cells that are in place) or monocytes (to mimic incoming cells at a later time point and also having a resident macrophage-like phenotype induced by the process of encapsulation as previous studies suggested) (Cha et al., 2017). In order to deconvolute the basis of interactions, co-culture systems with each component was also developed.

An interesting study done by Kirkpatrick and group previously, demonstrated that PMA treated THP-1 cells expressed macrophage markers such as CD68 and showed increased expression of macrophage related markers such as IL-1β, IL-8, and IL-10 secretion (Dohle et al., 2014). Previous transcriptomics studies also demonstrated that, under PMA activation THP-1 cells have a gene expression pattern similar to differentiated macrophages (Daigneault et al., 2010). Dohle et al. (2014) demonstrated that in three culture conditions (co-culture of osteoblasts, endothelial cells, and macrophages) the addition of THP-1 derived macrophages significantly increased the secretion of IL-6, IL-8, and TNF-α, which is in line with our observations. The main difference is that, in our model the 3-D ECM microenvironment was also mimicked by a crosslinked gelatin hydrogel based co-encapsulation of the cells which further guided the cell-cell interactions and provided sequestration of the secreted cytokines and their longer retention in a manner similar to the ECM.

## Conclusion

Macrophages are important actors in the host's reaction to implantable materials. In cell-based therapies, particularly in tissue engineering applications, their activities in the form of cytokine secretion, cell-cell contacts, and cross-talk with other cell types can be harnessed for faster maturation and better vascularization of artificial tissues with less pro-inflammatory responses. The effect can be pronounced by inducing further phenotypic control by incorporating specific stimulations for controlling macrophage phenotype. In this study, we have demonstrated that a combination of anti-inflammatory cytokines and macrophages can be used for improving the remodeling of hydrogels. The presence of IL-4 induced fibroblast proliferation in fibroblast/macrophage co-cultures resulted in the boost of cytokine secretion. The presence of monocytes/macrophages with IL-4 in co-culture conditions with endothelial cells resulted in more sprout-like organization within the hydrogel. When all three cell types were incorporated, the remodeling and population of the hydrogel structure together with the release of cytokines were improved. These results demonstrated the potential advantages of immune cells and immunomodulatory cytokines within tissue engineering context for better remodeling and vascularization. The presence of the immune component in this manner can also improve the fidelity of model organs for organ-on-a-chip applications. Our future work will focus on the demonstration of the effect of macrophage incorporation in specific organs and determination of the optimal macrophage phenotypes for different organ maturation.

## Author contributions

JB, CD, CM, and UL did the experiments. JB and NV designed the experiment. JB, NV, HK-M, and AD-B contributed to the interpretation of the results and the writing of the paper.

### Conflict of interest statement

JB, CM, and NV are full-time employees of Protip Medical. CD is an ex-employee of Protip Medical. NV is a shareholder of Protip Medical. UL is a full-time employee of Protobios. The collection, analysis, and interpretation of results presented was not influenced by the aforementioned companies. The remaining authors declare that the research was conducted in the absence of any commercial or financial relationships that could be construed as a potential conflict of interest.
