# Corrigendum: Immune Assisted Tissue Engineering via Incorporation of Macrophages in Cell-Laden Hydrogels Under Cytokine Stimulation

**DOI:** 10.3389/fbioe.2019.00029

**Published:** 2019-02-25

**Authors:** Julien Barthes, Camille Dollinger, Celine B. Muller, Urmas Liivas, Agnes Dupret-Bories, Helena Knopf-Marques, Nihal E. Vrana

**Affiliations:** ^1^PROTiP Medical, Strasbourg, France; ^2^INSERM UMR 1121, Strasbourg, France; ^3^Protobios LLC, Tallinn, Estonia; ^4^Institut Claudius Regaud, Institut Universitaire du Cancer Toulouse-Oncopole, Toulouse, France; ^5^Faculté de Chirurgie Dentaire, Université de Strasbourg, Strasbourg, France

**Keywords:** macrophage, encapsulation, hydrogels, tissue engineering, cytokines, co-culture

In the original article, there was a mistake in Figure 6 as published. At the bottom right, “BJ” was written instead of “HUVEC” for all the conditions. The corrected Figure 6 appears below.

**Figure 6:**
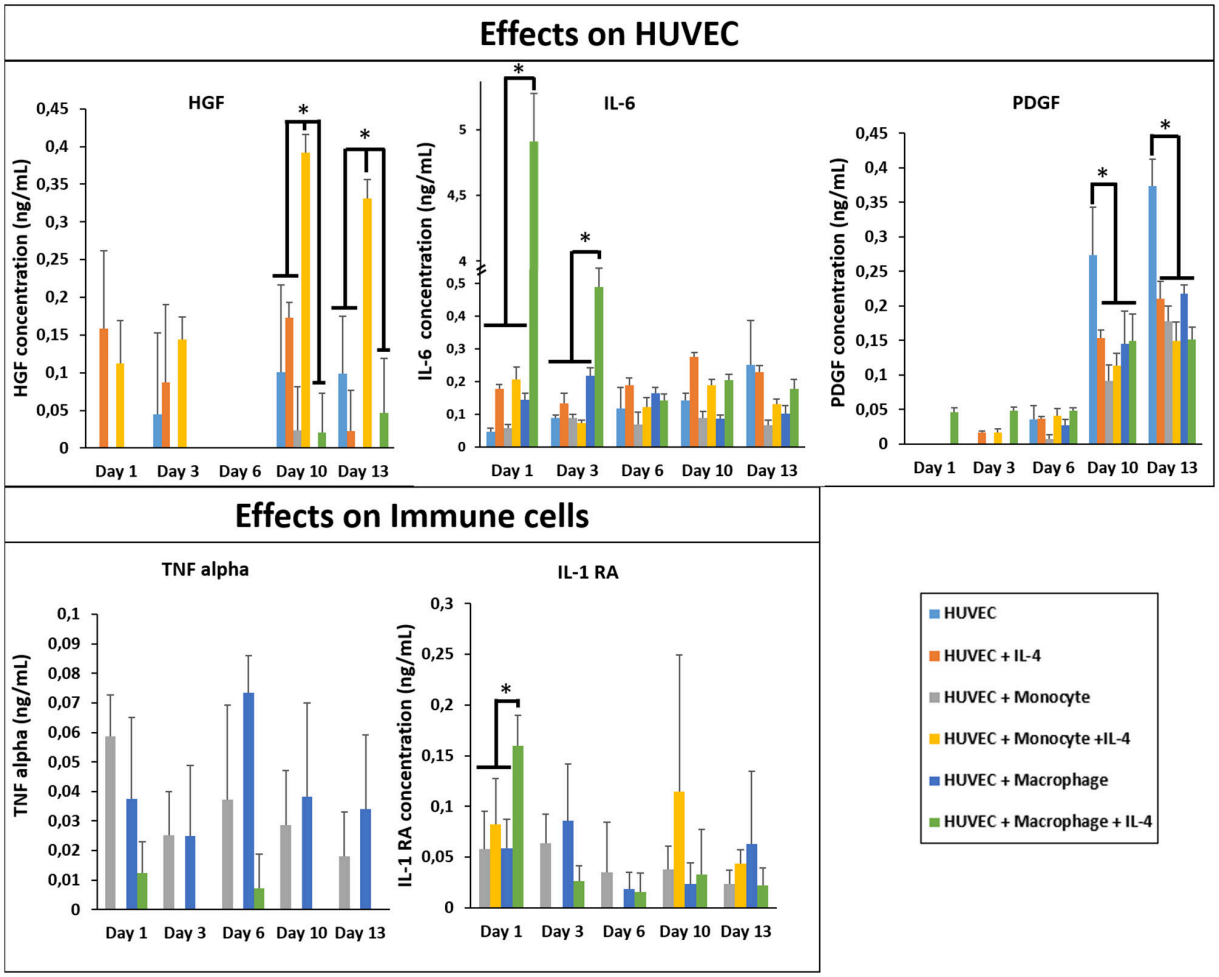
Cytokine quantification (HGF, PDGF, IL-1RA, IL-6, and TNF-α) in the supernatant at different time point for the co-culture experiments with and without IL-4 supplementation (*n* = 3) (*p < 0.05).

The authors apologize for this error and state that this does not change the scientific conclusions of the article in any way. The original article has been updated.

