# Supplementary material for: Immune Assisted Tissue Engineering via Incorporation of Macrophages in Cell-Laden Hydrogels Under Cytokine Stimulation
Source: Front Bioeng Biotechnol. 2018 Aug 20;6:108. doi: 10.3389/fbioe.2018.00108 (PMC6110199; doi:10.3389/fbioe.2018.00108)
Supplement: Supplementary file 1 [file Table_1.docx]

Supplementary Material

IMMUNE ASSISTED TISSUE ENGINEERING VIA INCORPORATION OF MACROPHAGES IN CELL-LADEN HYDROGELS UNDER CYTOKINE STIMULATION

Julien Barthes, Camille Dollinger, Celine Muller, Urmas Liivas, Agnes Dupret-Bories, Helena Knopf-Marques, Nihal Engin Vrana^*^

*** Correspondence:** Corresponding Author:


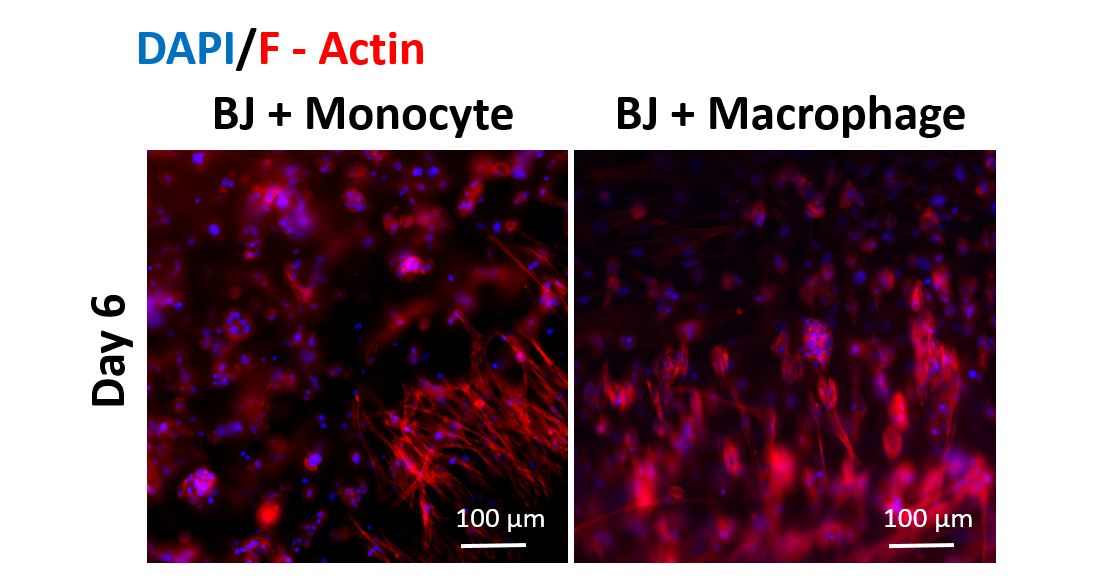


**Supplementary Figure 1.** Pictures obtained with epifluorescent microscope of encapsulated fibroblasts with either macrophage or monocyte with IL-4 supplementation stained with DAPI/Phalloidin.


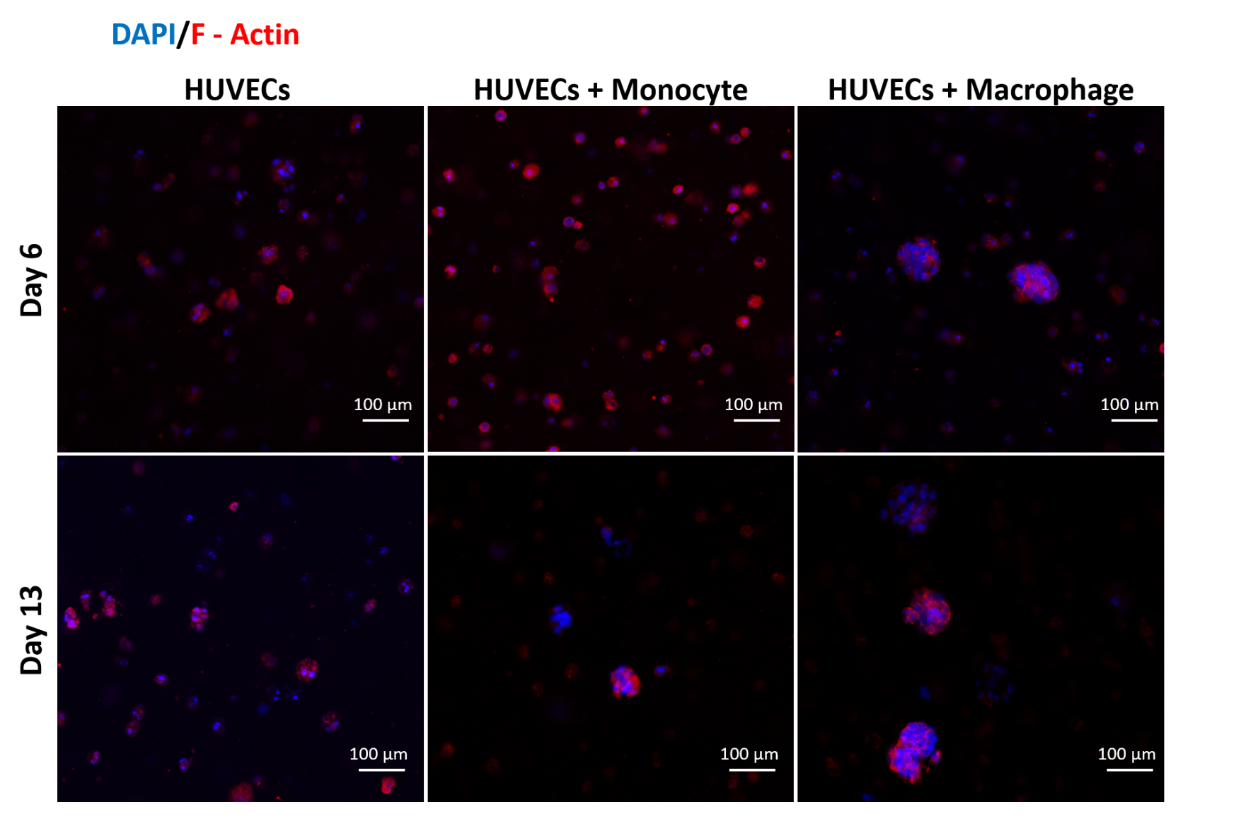


**Supplementary Figure 2.** Pictures obtained with confocal microscopoe microscope of encapsulated HUVECs with either macrophage or monocyte without IL-4 supplementation stained with DAPI/Phalloidin.


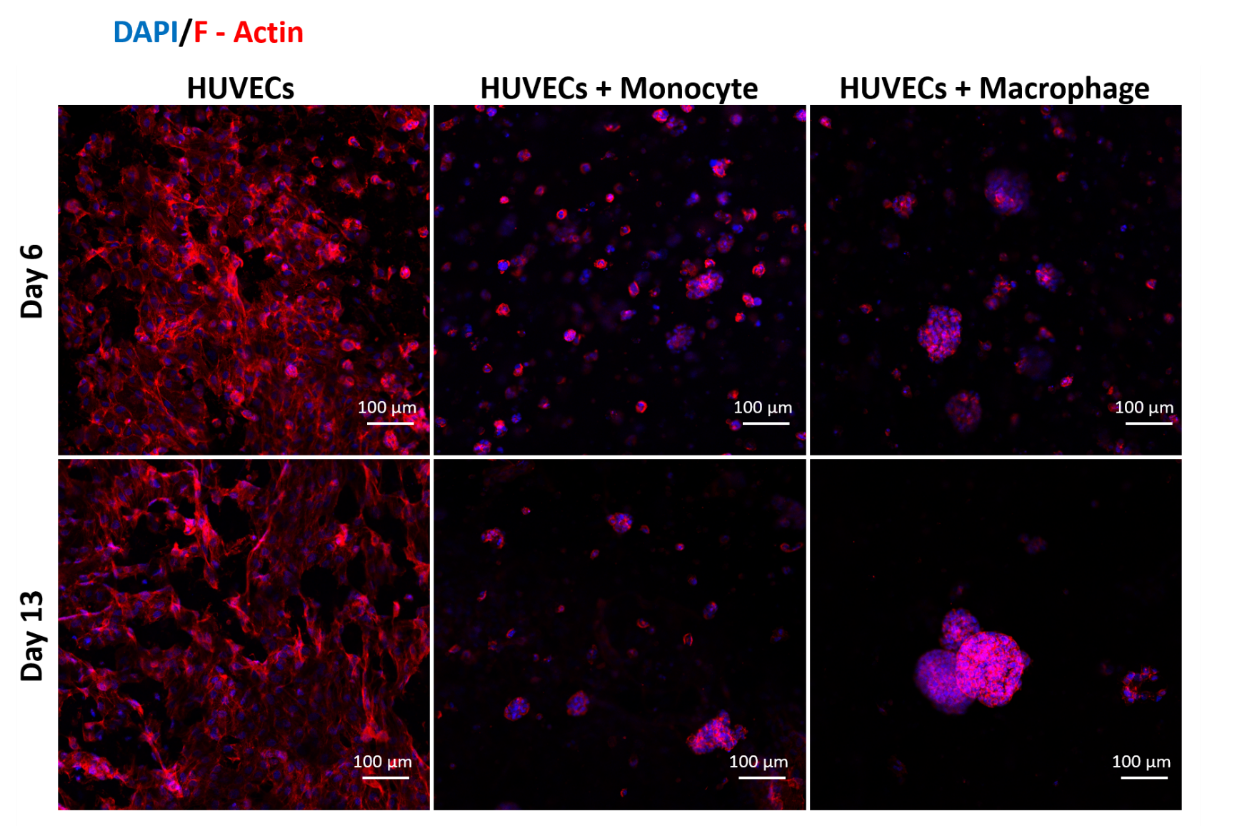


**Supplementary Figure 3.** Pictures obtained with confocal microscope of encapsulated HUVECs with either macrophage or monocyte with IL-4 supplementation stained with DAPI/Phalloidin.
